# Adaptation of plateau frog peptide: From antimicrobial to angiogenic and proliferative functions

**DOI:** 10.1016/j.jare.2025.06.013

**Published:** 2025-06-07

**Authors:** Kaixun Cao, Liting Zhang, Min Yang, Jinai Gao, Congshuang Deng, Xiaoshan Huang, Qian Chen, Qiumin Lu, Yizhe Cheng, Shaoyang Gao, Hui Cao, Ren Lai

**Affiliations:** a Key Laboratory of Agricultural Environmental Microbiology, Ministry of Agriculture and Rural Affairs, College of Life Sciences, Nanjing Agricultural University, Nanjing 210095, China; b Engineering Laboratory of Peptides of Chinese Academy of Sciences, Key Laboratory of Bioactive Peptides of Yunnan Province, KIZ-CUHK Joint Laboratory of Bioresources and Molecular Research in Common Diseases, National Resource Center for Non-Human Primates, National Research Facility for Phenotypic & Genetic Analysis of Model Animals (Primate Facility), State Key Laboratory of Genetic Evolution & Animal Models, Sino-African Joint Research Center, New Cornerstone Science Laboratory, Kunming Institute of Zoology, Chinese Academy of Sciences, No.17 Longxin Road, Kunming, Yunnan 650201, China; c Shenzhen Academy of Environmental Sciences, Shenzhen, Guangdong 518022, China; d State Key Laboratory of Quality Research in Chinese Medicine and Institute of Chinese Medical Sciences, University of Macau, Macao 999078, China; e Novatide (Kunming) Biotechnology Co., Ltd., Kunming, China; f Yunnan Characteristic Plant Extraction Laboratory Co., Ltd., Yunnan 650106, China

**Keywords:** Amphibians, EGFR, Wound healing, Angiogenesis, Purifying selection

## Abstract

**Introduction:**

Amphibian skin peptides, particularly defensins, play important roles in environmental adaptation, but often exhibit functional redundancy. SC17-2, a novel peptide from the high altitude frog Nanorana parkeri, exhibits unique angiogenesis and cell migration promoting activities, allowing adaptation to the extreme environment of the Tibetan Plateau with high UV radiation and low microbial diversity.

**Objectives:**

This study aimed to investigate the adaptive role of SC17-2 in high-UV environments, its functional differences from typical defensins, and its potential biomedical applications in wound healing and angiogenesis.

**Methods:**

Bioinformatics analyses, including sequence alignment and ancestral reconstruction, identified positively selected amino acid sites in SC17-2. Molecular docking examined its interaction with the epidermal growth factor receptor (EGFR). *In vitro* and *in vivo* experiments, using mouse and zebrafish models, assessed its wound healing and angiogenic properties.

**Results:**

SC17-2 exhibited no antimicrobial activity, but it demonstrated antioxidant activity and potent wound healing and angiogenic properties. Molecular docking indicated that SC17-2 interacts with EGFR, potentially activating downstream signalling pathways. *In vivo* experiments showed that SC17-2 significantly accelerated wound healing by promoting collagen regeneration and angiogenesis, in some aspects outperforming VEGF.

**Conclusion:**

SC17-2 represents a unique functional divergence in amphibian peptides, driven by ecological adaptation rather than microbial pressure. Its ability to promote angiogenesis and cell migration highlights its potential as a novel therapeutic agent for regenerative medicine, shaped by the extreme conditions of the Tibetan Plateau.

## Highlights

• SC17-2 from plateau frog lacks antimicrobial activity but drives angiogenesis and wound healing.
• Dual EGFR conformation binding activates MAPK/Smad pathways for tissue repair and vascular growth.
• Ecological UV/cold pressures, not microbes, shape functional divergence of amphibian peptides.
• SC17-2 outperforms VEGF in collagen regeneration and accelerates wound closure in murine models.
• Purifying selection enables structural adaptation for EGFR interaction in high-altitude environments.
• Integrates evolutionary biology and biomedicine to redefine peptide roles in ecological adaptation.

## Graphical abstract

**Figure:**
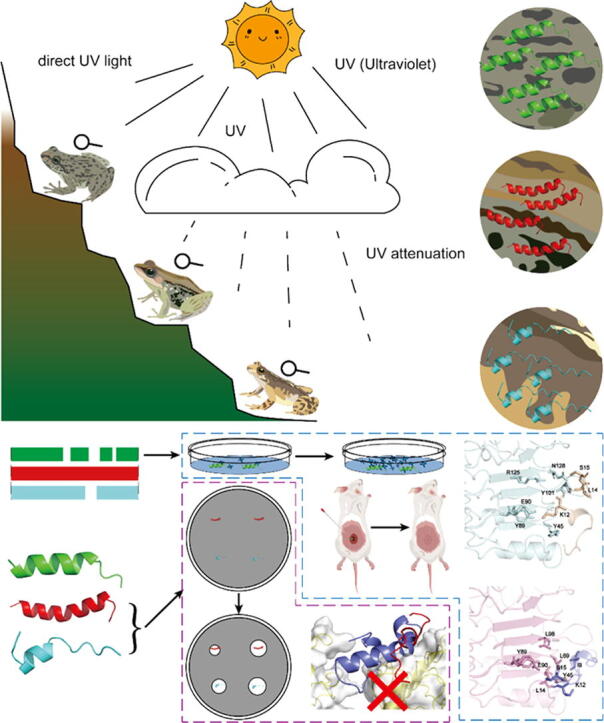

## Introduction

The Ranidae family, commonly known as true frogs, is distributed across diverse ecosystems worldwide [1]. These frogs can be found in diverse environments, including tropical lowlands [2] and high-altitude mountains [3]. Despite their relatively exposed and fragile skin [4,5], these frogs have demonstrated remarkable adaptability [3,6,7], largely due to the abundance of bioactive compounds in their epidermis. Previous studies have identified a significant number of peptides that show considerable potential in the field of drug development. Of particular interest are antimicrobial peptides (AMPs) [8], including defensins, antioxidant peptides [7], and tissue repair peptides [9]. It is typical of frogs to secrete multiple peptides simultaneously, resulting in synergistic effects that enhance survival [10]. The diversity and abundance of these peptides are often correlated with their habitats, suggesting that environmental heterogeneity may be a driver of frog evolutionary adaptation [6,11,12]. Pleskein is a class of AMPs that has been extracted from *N. pleskei* [7,13], a species that is distributed in highland marshes at elevations of 3300–4500 m. The pleskein family of AMPs has demonstrated antimicrobial and antioxidant activities, which may be adaptations to high-altitude environments. *Rana sylvatica* experiences low ambient temperatures in winter, which induces the secretion of brevinin-1 family AMPs [14].

Frogs are exposed to a wide range of aquatic and terrestrial pathogens during the tadpole to adult stages, and thus the skin is enriched with diverse AMPs to counteract a wide range of infectious threats [[15], [16], [17]]. AMPs, as the first line of defence against microorganisms in frogs, are the most frequently reported active peptides [15,18], and several superfamilies of AMPs such as Brevinin [19], Temporins [20], and Japonicin [21] have been reported to date. These AMPs possess a distinctive attribute: a highly conserved signal peptide portion and a diverse mature peptide portion [22,23]. The conservation of the signal peptide portion can be indicative of its motif origin [24], while the diversity of mature peptides can facilitate a rapid host response and adaptation to the environment [25,26]. Despite the considerable variation in the mature sequences of different AMPs, they demonstrate similarities within the AMP family, including amphiphilic arrangement and specific motifs (e.g. Trp is involved in membrane anchoring, Pro affects the selectivity of membrane interactions in the region) [10,27], as well as the structure of the disulfide ring known as the 'Rana box' [28]. The analysis of these regions of similarity and results has been facilitated by the 'Rana box' [29,30].

It is widely recognized that these AMP variants have undergone convergent evolution, acquiring novel functions through adaptive modifications [31]. Tigerinins from *R. tigerina*, for instance, have been shown to possess wound-healing and anti-diabetic properties in addition to their established antibacterial activity [32]. Furthermore, AMPs in frogs have been observed to exhibit cytolytic activity, functioning in synergy with neuropeptides to elicit anti-predator effects [33]. Some AMPs, including Temporins A and B, have been demonstrated to activate the epidermal growth factor receptor (EGFR) and its downstream signalling pathways, while concomitantly scavenging microorganisms, thereby promoting wound healing [34]. Transforming growth factor-α (TGF-α) and epidermal growth factor (EGF) have been shown to be structurally related [35], and both have been demonstrated to be influenced by the common receptor, EGFR [36]. It has been reported that EGFR can modulate the expression and regulation of EGF or TGF and their downstream signalling pathways through small conformational changes [37]. The induction of EGF or TGF-α has been shown to result in elevated mRNA expression of TGF-α and EGF [38,39], which in turn have been observed to promote cell proliferation, migration and angiogenesis by affecting other downstream signalling pathways. For instance, Smad2/3 signalling activity has been demonstrated to promote endothelial-mesenchymal transition and vessel wall regeneration [40]. In addition, TGF-β and other pro-fibrotic factors have been shown to promote the transformation of myofibroblasts [41]. However, it should be noted that TGF-β does not promote fibroblast proliferation; rather, it induces MAPK signalling to promote cell proliferation and migration [42]. This mechanism may underpin the observed effects of certain frog-derived AMP peptides on wound healing and angiogenesis [34,43].

*Nanorana parkeri* (*Nanorana parkeri Stejneger*, L., 1927.) is a species of amphibian endemic to the Tibetan Plateau, where they have evolved stronger UV-resistant, antioxidant, and other high-altitude survival strategies to adapt to the Tibetan Plateau environment [44]. In this study, we report a pleskein family antimicrobial peptide (AMP), designated SC17-2, which was derived from the frog skin transcriptome. This peptide did not exhibit antimicrobial activity, but instead demonstrated antioxidant, wound repair, and pro-angiogenic activities. In the subsequent phase of the study, the positive selection and ancestral origins of SC17-2 and other AMPs with high homology in Ranidae were investigated. This research may offer novel insights into the evolutionary strategies employed by Ranidae-derived peptides in environmental adaptation.

## Materials and methods

### Materials

Human immortalized keratinocytes (HaCaT), HSF (Human Skin Fibroblast), HUVEC (Human Umbilical Vein Endothelial Cells), *Escherichia coli* (ATCC2771), *Acinetobacter baumannii* (ATCC 19606), *Candida auris* (ATCC10231) and *Staphylococcus aureus* (ATCC 6538) were purchased from ATCC (USA). BALB/c mice (all 6 weeks, male) were purchased from Charles River Laboratories (Beijing, China). H&E, dewaxing solution, Masson and Anti-CD31 Mouse mAb were purchased from Servicebio (Wu Han, China). Transgenic zebrafish Tg (*Fli1a:eGFP*) was kindly provided by the State Key Laboratory of Quality Research in Chinese Medicine (University of Macau). Cell Counting Kit-8, Phosphatase Inhibitor Cocktail II, Protease Inhibitor Cocktail and VEGF165 Protein, Human (HEK293, C-His), EGF Protein, Human and 5X All-In-One MasterMix and BlasTaq 2X MasterMix were purchased from MedChemExpress (Shanghai, China). phosphate buffer solution (PBS) was purchased from Corning (USA). SDS-PAGE Protein Sampling Buffer (5X) was purchased from Biosharp (Hefei, China). Goat Anti-Rabbit IgG (H + L) HRP was purchased from Affinity Biosciences (USA). CST 4370 T Phospho-p44/42 MAPK(Erk1/2) (Thr202/Tyr202/Tyr202) was purchased from Abcam (UK). (Thr202/Tyr204) (D13.14.4E) XP Rabbit mAb, CST 4695 T p44/42 MAPK(Erk1/2) (137F5) Rabbit mAb, CST 8828S Phospho-Smad2(Ser465/467)/Smad3(Ser423/ 425)/Smad3(Ser423/(D27F4) Rabbit mAb were purchased from Cell Signaling (USA). smad2/3 Rabbit pAb was purchased from ZEN BIO (Chengdu, China). pageRuler Prestained Protein Marker, 10–180 kDa was purchased from Thermo Scientific (USA). DMEM/F12 was purchased from cellgro (USA). The fetal bovine serum, FBS, was purchased from Gibco (Thermo Fisher Scientific, USA), and primer synthesis was performed by Sangon (Shanghai, China).

### Peptide synthesis

Following a thorough positive selection analysis of the peptide sequences, the fragment exhibiting the most significant difference was identified: SFMPLSIPIMCKMLSKC and named SC17-2. Peptide SC17-2 was synthesised by Gotopbio (Hangzhou, China) via solid-phase synthesis. Cyclic reduced peptides were purified by reversed-phase HPLC (RP-HPLC). Fractions of high (<95 %) HPLC homogeneity and with expected mass were combined, pooled and lyophilized (supplementary material Fig. S1).

### Ethics statement

All animal care and experimental procedures were approved by the Ethics Committee for Laboratory Animal Welfare, Kunming Institute of Zoology, Chinese Academy of Sciences (IACUC-RE-2024-08-011). The zebrafish experiments were approved by the Animal Research Ethics Committee, University of Macau (approval number: UMARE-021b-2020).

### Transcript assembly and quantitative analysis

Quality control was conducted using FastQ [45] and FastQC [46], followed by transcript assembly with RNA-Bloom [47]. Transcript translation was then performed with TransDecoder [48]. Subsequently, 1690 sequences were downloaded from UniProt [49] using the keywords “frog” and “antimicrobial”. Thereafter, Diamond [50] “-blastp” was employed to identify homologous analogues of frog AMP, and these protein sequences were subsequently obtained with seqkit [51].

### Multiple sequence comparison and pairwise comparison

The homologous protein sequence of SC17-2 was obtained via NCBI BLASTP [52], which was subsequently aligned using Quick Tcoffee [53] alignments set in JalView [54]. The amino acid and nucleotide sequences were processed following a Tcoffee comparison using PAL2NAL [55] in order to obtain codon alignment results. The optimal evolutionary tree was then constructed using IQ-TREE [56], and site selection was analysed using HyPhy's [57] fixed effect likelihood (FEL).

### Antibacterial test *in vitro*

Disk diffusion and broth microdilution tests were selected as the standard method for screening the antimicrobial activity of SC17-2 [58]. The experiments were conducted utilising the disk diffusion test, whereby the following strains were examined: *Acinetobacter baumannii* (ATCC 19606), *Candida auris* (ATCC 10231), *Pseudomonas aeruginosa* (ATCC 27853) and *Staphylococcus aureus* (ATCC 6538). The concentration of bacterial suspension was then adjusted to 1 × 10^5^ CFU/mL with saline, after which it was set aside. Separate plates containing bacterial agar were prepared: MH agar was utilised for the isolation of bacterial samples, while Sartorius agar was employed for the isolation of fungal samples. The suspensions containing the aforementioned microorganisms were then spread on the designated plates at a rate of 1:1000. A sterile filter paper was used to dispense 1 mL of SC17-2 (10 mg/mL), with equal amounts of PBS and Vancomycin/Colistin serving as controls. The plates were then subjected to the appropriate temperature (24 h at 37 °C) and subsequently observed under a camera to ascertain the results.

A total of 100 μL of each of the following bacterial strains was added to 24-well plates: *Acinetobacter baumannii* (ATCC 19606), *Escherichia coli* (ATCC2771) and *Staphylococcus aureus* (ATCC 6538). Following this, the SC17-2 was incorporated into the mixture at concentrations of 0, 3.125, 6.25, 12.5, 25, 50, and 100 μg/ml. The positive control was established by utilising equal volumes of vancomycin and colistin. Subsequently, the mixtures were incubated at 37 °C for 24 h. At the conclusion of this period, the absorbance was measured using a Microplate Reader (Synergy H1, BioTek, USA) with a wavelength of 600 nm. The bacterial solution, prepared without the addition of an equal volume of saline, served as the control.

### *In vitro* cytotoxicity assay

Following the same procedure as in the previous HaCaT, HSF and HUVEC cell cytotoxicity assays [59], the cells were seeded in 96-well plates at a density of 1 × 10^4^ cells/well and treated with various concentrations of SC17-2 for 24 h, the absorbance was measured using a MicroplateReader (SynergyH1, BioTek, USA) at a wavelength of 450 nm, and the control group was treated with an equal volume of saline and all results were normalised.

### *In vitro* UVB damage repair

HaCaT cells were inoculated in 24-well plates at 1 × 10^4^ cells/well and incubated for 24 h to ensure uniformity and number of cells per well. Prior to UVB irradiation, cells were washed with phosphate buffer solution (PBS) and covered with 300 μL of PBS. The blank control group was wrapped through tinfoil to eliminate the effects caused by UVB irradiation and subsequently irradiated through 5 J/cm. Immediately after irradiation, PBS was replaced with DMEM/F12 medium and 20 and 5 μg/mL of peptides SC17-2 were added, respectively, and incubated for 24 h. After that, 10 μL of CCK-8 was added to each well, and incubated for 2 h. The absorbance values of each well were measured at 450 nm.

### Animal wound healing experiments

Mice were anaesthetised with sodium pentobarbital 2 % by intraperitoneal injection (0.1 mL/20 g mouse body weight), and after anaesthesia, hair was removed from the back using depilatory cream and power scissors, and circular full-thickness skin wounds of the same diameter were marked and created on the back of each mouse using circular stamps and red sealant. The mouse wounds were then covered and infiltrated daily with 20 μL of different concentrations of SC17-2 or 20 ng/mL VEGF, and an equal volume of saline was used in the control group. The mice were anaesthetised by isoflurane nebulisation (concentration 2 %) every two days and then placed under an optical camera to photograph the effect of the wound area.

### Masson and H&E staining

Sodium pentobarbital (2 %) was utilised for the anaesthetisation of mice by intraperitoneal injection (0.1 mL/20 g of mouse body weight). Subsequent to the induction of anaesthesia, the dorsal hair was removed using depilatory creams and electric scissors. Thereafter, circular full skin wounds of the same diameter were marked and created on the back of each mouse using circular stamps and red stencils. Mouse wounds were then covered and infiltrated daily with 20 μL of different concentrations of SC17-2 or 20 ng/mL of VEGF, and an equal volume of saline was used in the control group. Mice were anaesthetised every two days using isoflurane nebulisation followed by anaesthesia (concentration 2 %), after which they were placed under an optical camera to photograph the effect of wound area. After 13 days of treatment, mice were anaesthetised using isoflurane nebulisation followed by injection of an overdose of sodium pentobarbital to euthanise the mice (200 mg/kg). The wounds and their surrounding tissues were then excised for histopathological evaluation (Masson and H&E staining) and analysed by Enface immunofluorescence staining.

### Immunofluorescence

Use scissors to remove the tissue around the wound, put the tissue into 4 % paraformaldehyde solution, put the tissue in 4 °C for 24 h, use a tissue embedding machine for embedding, use a rotary slicer to section the tissue, put the section into environmentally friendly dewaxing solution to dewax for 3 times, 10 min each time, then use anhydrous ethanol to treat the section for 3 times, 5 min each time, rinse with distilled water and then repair the section by EDTA antigen repair solution. The slides were then incubated overnight at 4 °C with Anti-CD31 Mouse mAb as primary antibody and at room temperature for 50 min, protected from light, in PBS (pH 7.4), shaken and washed 3 times for 5 min each time on a decolourising shaker. Incubate at room temperature for 10 min. Images were captured using a scanner (Pannoramic MIDI), and graphics were processed using imageJ. The results were calculated as follows: relative staining area of blood vessels = A_n_/average (A_c_), A_n_ indicates the fluorescence staining area of CD31 obtained from the current sample by ImageJ statistics, and A_c_ is the area of all the control samples by ImageJ, and Ac is the fluorescence staining area of CD31 of all control samples.

### Proangiogenesis experiments in zebrafish

Transgenic zebrafish Tg (*Fli1a:eGFP*) were cultured in culture tanks at 28 °C in a 14 h:10 h light/dark cycle for up to 6 months and subsequently made to produce zygotes by mating only, zebrafish larvae at 24 h post fertilisation (hpf) were induced for 3 h with a VEGFR tyrosine kinase inhibitor II (VRI) to produce a model of vascular insufficiency [60], after which they were washed to abort the effect of VRI was added to the same number of zebrafish of different groups with SC17-2 or equivalent amount of PBS, and images of zebrafish embryos were captured by Leica fluorescence microscopy, and the ISV index was calculated as follows: ISV index = intact blood vessels × 1 + defective blood vessels × 0.5.

### Western blot

HaCaT was inoculated in 12-well plates (2 × 10^5^ cells/well) and cultured for 24 h. Afterwards, HaCaT cells were treated with equal amounts of VEGF, SC17-2, and PBS for 16 h. Cells were washed with PBS, after which the samples were collected and added to 1 mL of RIPA lysate containing protease inhibitors and phosphatase inhibitors and placed on ice for 30 min. Allowed to be after sufficient lysis, the supernatant was centrifuged and collected to determine the protein concentration using a nanodrop, followed by the addition of SDS sampling buffer and heating at 95 °C for 10 min. Samples were loaded and run on a gel block based on the determined sample concentration, and then electrotransferred onto a 0.45 μm PVDF membrane. The membrane was closed with 5 % skimmed milk and incubated with diluted primary antibodies (Smad2/3, p-Smad2/3, ERK, p-ERK, GAPDH, and TGF-β) overnight at 4 °C, followed by 1 h of incubation with the corresponding secondary antibodies. After washing three times with TBST, proteins were visualised and scanned by enhanced chemiluminescence using the ChemiDoc XRS imaging system (Bio-Rad, Hercules, CA, USA).

### Quantitative real-time PCR

HaCaT cells were treated as above, washed twice with PBS and lysed using Trizol for 5 min, after which 200 μL of chloroform was added and shaken vigorously for 30 s. The cells were centrifuged for 15 min to separate the upper and lower layers (12,500 g, 4 °C). The supernatant was vortexed with an equal amount of isopropanol and left to stand for 10 min, and then centrifuged again under the same conditions to retain only the white precipitate. 500 μL of 75 % ethanol was added and centrifuged (12000 rpm, 4 °C, 10 min) and the operation was repeated twice. Afterwards, DEPC was used for dissolution and the concentration was determined by nanodrop. One-step reverse transcription to DNA was performed using 5 × MIX, after which primers and 2 × MIX were added and placed. qPCR was performed (LightCycler96, F. Hoffmann-La Roche, Swiss).

### Bio-Layer Interferometry experimental (BLI)

Prepare 20 μg/mL of EGFR protein solution (solvent 0.1 % PBS-Tween), and different concentrations of SC17-2 (5, 10, 20 μg/mL) dissolved using PBST. Biotin was added and incubated at room temperature for 30 min according to the instructions, and the corresponding probes were soaked in PBST for 10 min. The assay was carried out according to the BLI predefined procedure (Octet K2, ForteBio, USA), followed by calculations using the accompanying software, and graphpad was used for graphing.

### Surface plasmon resonance (SPR)

The analysis of biomolecular interactions was conducted utilising a Biacore S200 biosensor system (S200, Biacore, China). Briefly, the EGFR (20 μg/mL) was immobilised by amine coupling on the surface of a CM5 sensor chip (29104988, Cytiva, China) with a resonance unit (RU) of approximately 2000, using a sodium acetate buffer (10 mM, pH 4) as the immobilisation solution. In order to assess the affinity of SC17-2 for the epidermal EGFR, SC17-2 was injected at a flow rate of 30 μL/min onto a chip containing immobilised EGFR. The injection of SC17-2 was carried out at a flow rate of 30 μL/min at concentrations ranging from 5 to 500 μg/mL. The RU were recorded. The binding dissociation constants (*KD*) were determined using BiacoreTM Insight software.

### Docking

To gain insight into the detailed binding conformations of the two reported conformations of SC17-2 and EGFR, docking was performed via the HADDOCK web server [61] (https://rascar.science.uu.nl/haddock2.4/; accessed: 2024-11-20). The active sites for both conformations of EGFR (PDB code: 3NJP) were reported according to Huang et al [37]. using the AlphaFold2 web service [62] (https://colab.research.google.com/github/sokrypton/ColabFold/blob/main/AlphaFold2.ipynb; accessed: 2024-7-17).

### *In silico* toxicity prediction

The prediction of toxicity for SC17-2 was conducted utilising the ToxinPred [63]. The prediction of hemolytic activity was predicted by HemoPI2.0 [64]. Furthermore, the potential allergenicity of SC17-2 was predicted through the implementation of AllerTOP [65].

### Statistical analysis

ImageJ was used to quantify the percentage of stained area for Masson and the percentage of fluorescent area for CD31. All data were analysed by GraphPad Prism 9.5 software. Data were expressed as mean ± SEM. Comparisons between multiple groups were performed by using one-way analysis of variance (ANOVA) and Tukey's multiple comparison test. Differences between groups were commonly tested by ANOVA, *P* < 0.05 was considered statistically significant.

## Results

### SC17-2 is an AMP in Ranidae

**Fig. 1:**
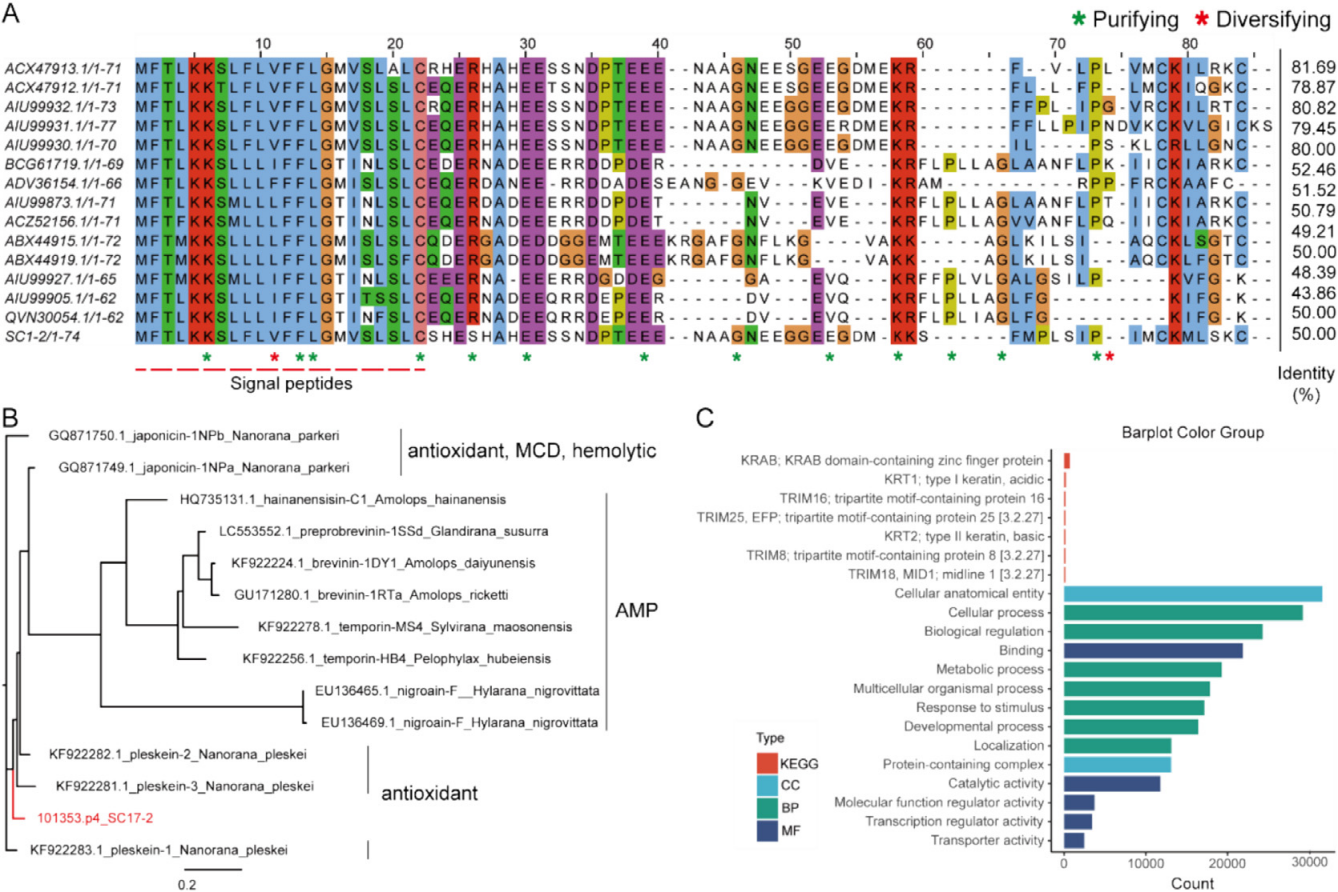
The multiple sequence comparison, ancestry reconstruction and metabolic pattern analysis of SC17-2 in the skin of the *N. parkeri*. (A) SC17-2 and its family of homologous proteins are AMPs, and the full-length sequence of their signal peptide portion is indicated by a red dashed line, with red asterisks denoting Diversifying selection sites and green asterisks denoting Purifying selection sites. (B) Ancestry reconstruction of SC17-2 and its homologous sequence demonstrated that SC17-2 was, in fact, an early isolate of the antimicrobial peptide family. (C) Enrichment analysis revealed a considerable number of metabolic processes and interactions with environmental stimuli in frog skin. Additionally, KEGG pathway analysis indicated the presence of both transcriptional regulation and regulatory processes. (For interpretation of the references to colour in this figure legend, the reader is referred to the web version of this article.)

**Fig. 2:**
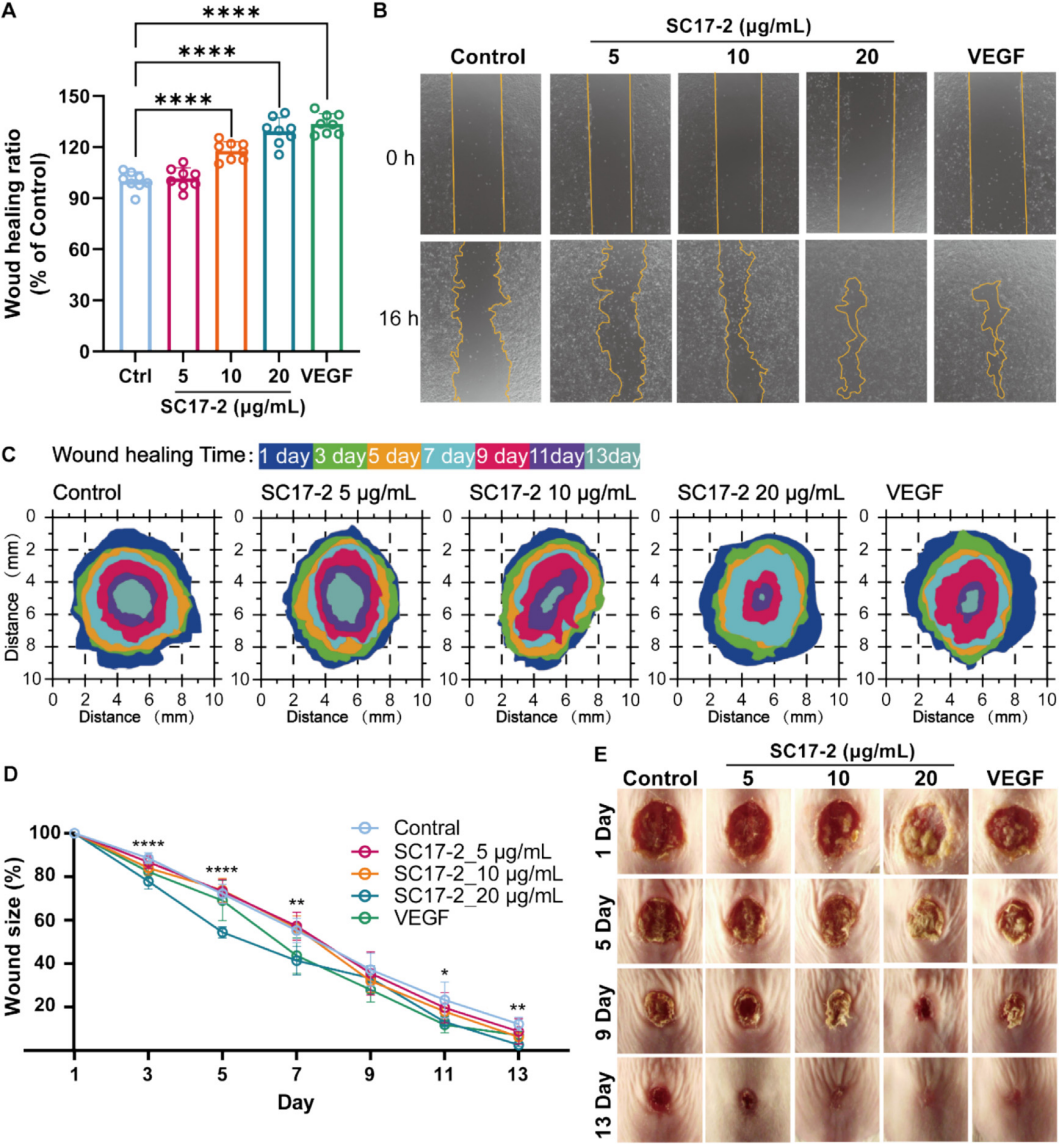
Results of the cell scratch experiment and mouse wound healing experiment with different concentrations of SC17-2. (A) After 16 h of different treatments, the repair rate of HaCaT cell scratches showed that there were significant differences between the 10 μg/mL, 20 μg/mL concentration treatments of SC17-2 and the VEGF treatments compared with Control treatment (n = 8). (B) The representative migration images of the HaCaT after treated with or without increasing concentration of SC17-2 for 16 h, the group receiving 20 μg/mL VEGF was set as normal control. (C and E) The representative images of the wound area of mice under different treatments (n = 7). (D) The quantification of the wound area of mice under different treatments. (Compared with the control group: * *P* ≤ 0.05, ** *P* ≤ 0.01, and *** *P* ≤ 0.001.).

As demonstrated in Fig. 1, we employed local BLASTP to identify reported homologous analogues of AMP in Ranidae with peptide SC17-2 from *N. parkeri*. The red dashed line indicates the signal peptide. The effect likelihood (FEL) of HyPhy [57] was then utilised for the detection of positively selected codons, which revealed 13 purifying sites and 2 diversifying sites. Given the diversity of amino acid positional differences after amino acid 59, amino acids 59–84 were then intercepted for subsequent experiments. Ancestral reconstruction of these peptides by ancseq [66] (see Fig. 2) reveals that SC17-2 is located in a topological position between branches of the pleskein family, suggesting that SC17-2 belongs to a member of the broad AMP family of Ranidae, which is more closely related to the pleskein family that has been reported to have antioxidant activity. Furthermore, KEGG enrichment analysis of the transcriptomes of the samples showed that transcriptional regulation and transcriptional regulatory processes were active in the skin of the *N. parkeri*, which is consistent with the environmental requirements of this species [67]. However, it is important to note that the result of paper-disc agar-diffusion method and MIC of SC17-2 indicates that the peptide does not appear to have antimicrobial activity (Table S1 and Fig. S3). Circular dichroism spectroscopy revealed that the secondary structure of SC17-2 remained largely unchanged when exposed to either 60 mM or 0 mM SDS. Both samples consisted primarily of random coil and β-sheet structures, with only trace amounts of γ-turn and α-helix structures present (Fig. S2).

### SC17-2 promotes cell migration and wound healing in mice

We used HaCaT HSF and HUVEC cells to determine whether SC17-2 could promote cell migration based on past reports of pleskein family activity (Fig. S5). As illustrated in Fig. 2A, the *in vitro* wound healing assay of SC17-2 demonstrated that treatment at concentrations exceeding 10 μg/mL for 16 h resulted in a notable enhancement of HaCaT, HSF and HUVEC migration. This result was corroborated by the mouse wound healing assay, in which 20 μg/mL of SC17-2 was sufficient to significantly promote the reduction of wound area at times other than day 9 (Fig. 2C and Fig. 2D). Specifically, mice treated with 20 μg/mL of SC17-2 on days 3 and 5 exhibited the most rapid reduction in wound area, with a mean of 77.84 ± 3.34 % and 54.43 ± 2.53 % (Fig. S6), respectively, which was significantly less than that of the control group. These values were found to be almost 1.3-fold higher compared to the saline-treated control group. Furthermore, the non-significant difference observed on day 9 may be attributed to an increase in wound area due to crusting (Fig. 2E). Following a 13-day treatment period, the final reduction in wound area was found to be metrologically dependent on SC17-2. Mice treated with 20 μg/mL of SC17-2 exhibited a wound area of 2.65 ± 0.72 %, which was significantly smaller than that observed in mice treated with VEGF (7.04 ± 1.43 %).

### SC17-2 promotes collagen regeneration and granulation in *peri*-wound tissues

**Fig. 3:**
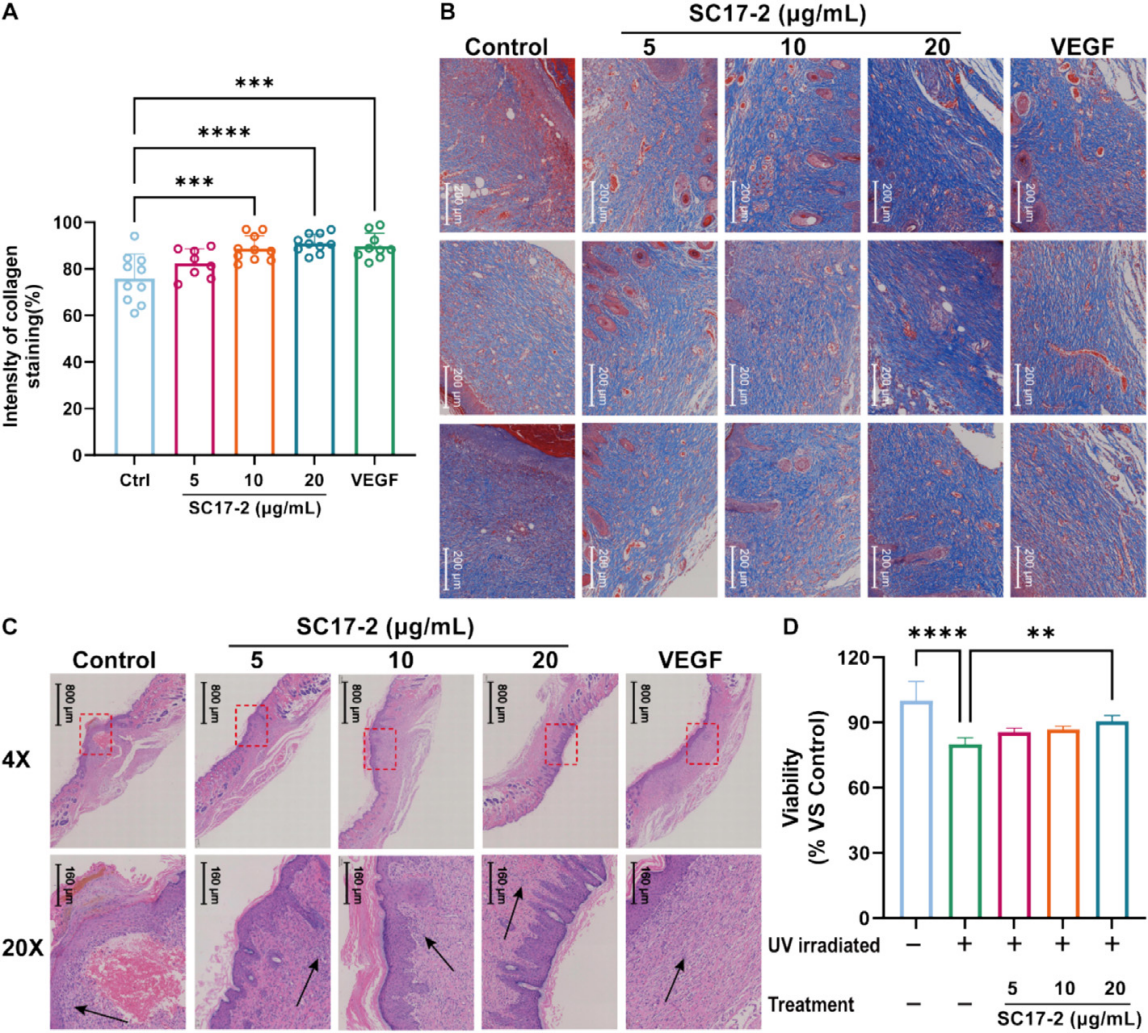
Masson and H&E staining analysis of mice wound sections after treatment with different concentrations of SC17-2 at day 13. (A) The collagen staining intensity showed that there were significant differences between the 10 μg/mL and 20 μg/mL concentration treatments of SC17-2 and the VEGF treatment in comparison with the Control treatment (n = 10). (B) The Masson staining images of the wound sections in mice after 13 days of the different treatments. (C)The H&E staining images of the wound area showed that SC17-2 significantly reduced the degree of inflammatory cell infiltration in the wound tissue when the concentration of SC17-2 no less than 10 μg/mL, similar to that of VEGF. The black arrows indicate the presence of inflammatory cells. (D) The application of SC17-2 could attenuate the cell injuries of HaCaT after exposed to UVB irradiation (n = 3). (Compared with the control group: * *P* ≤ 0.05, ** *P* ≤ 0.01, *** *P* ≤ 0.001, and **** *P* ≤ 0.0001.).

A comparison of the relative effects of different treatments on collagen regeneration and granulation in wound tissue on day 13 by Masson and H&E staining, and ImageJ statistics of the intensity of the blue staining revealed that SC17-2 at concentrations above 10 μg/mL exhibited a similar capacity to VEGF in significantly enhancing collagen regeneration (Fig. 3A, Fig. 3B).

As illustrated in the H&E staining image in Fig. 3C, SC17-2 also demonstrated a notable impact on attenuating the inflammatory response in wounds. An increase in the concentration of SC17-2 was associated with a notable thickening of epithelial tissue and enhanced granulation tissue formation. Furthermore, the capacity of SC17-2 to eliminate UVB-induced damage was also evaluated. The results demonstrated that a concentration of 20 μg/mL of SC17-2 was capable of effectively removing the damage caused by UVB radiation, when compared to a control group in which an equal volume of saline was administered following irradiation. However, the effect was found to be considerably less pronounced than that observed in the treatment group that had not been subjected to irradiation.

### SC17-2 promotes angiogenesis in mice and zebrafish

**Fig. 4:**
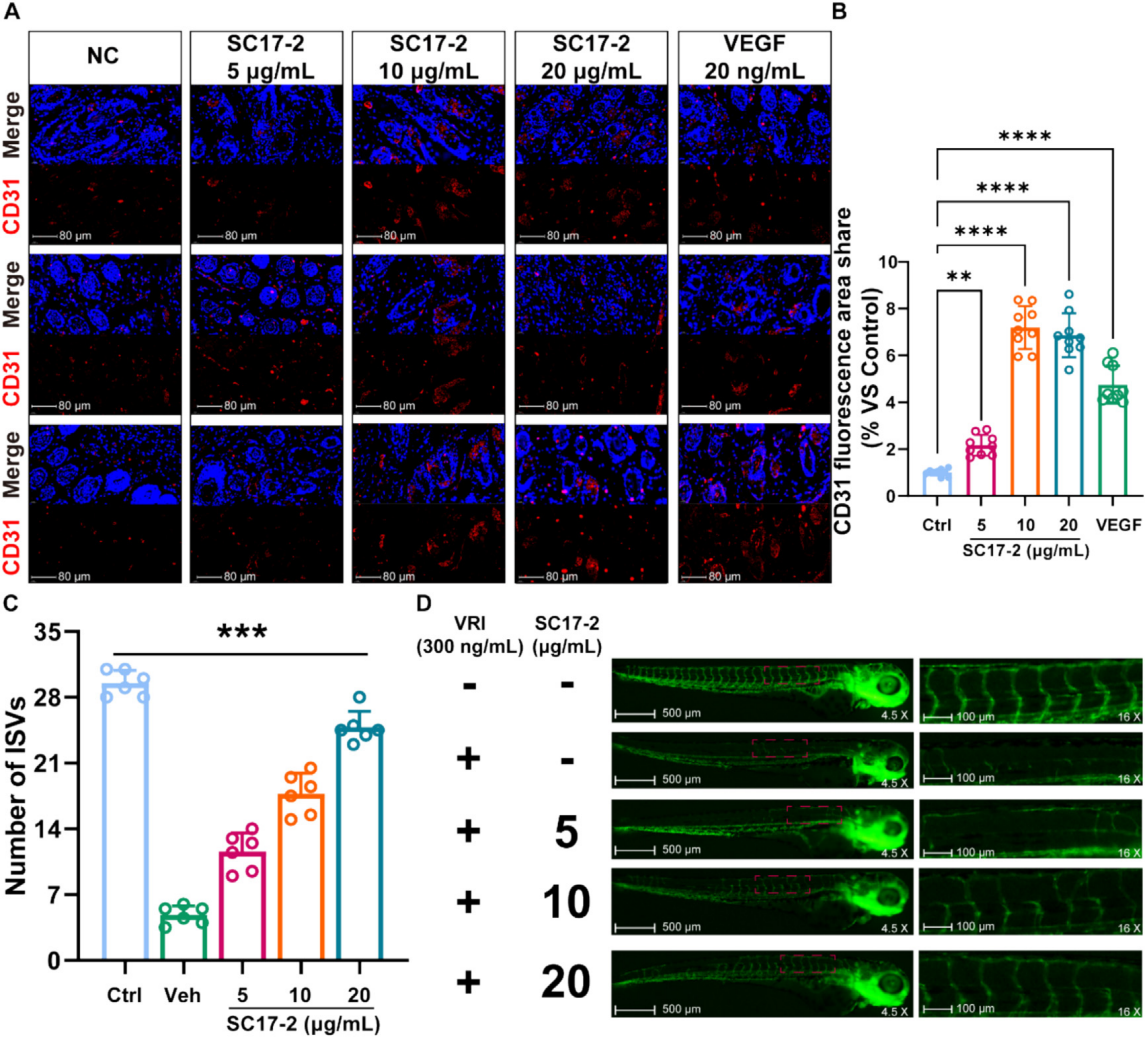
SC17-2 promotes angiogenesis in mice and zebrafish *in vivo*. (A) Immunohistochemical images of vascular endothelium labelled with CD31 in wound tissue sections of mice after 13 days of five treatments showed that the number of blood vessels in the wounds increased with the increase of SC17-2 concentration. Where, blue is the result of DAPI staining and red is the result of CD31 staining. (B) Comparison of the fluorescence staining intensity of CD31 in mouse wound tissue sections after 13 days of five treatments showed that SC17-2 could effectively promote angiogenesis (n = 9). (C) Administration of SC17-2 was able to effectively alleviate the loss of blood vessels in zebrafish embryos after the induction of VRI (n = 6). (D) Representative images demonstrating control and intersegmental vascular (ISV) growth in VRI (300 ng/mL)-treated zebrafish embryos and the results of incubation at 24 hpf for 24 h with or without SC17-2 (5, 10, 20 μg/mL). (Compared with control group: * *P* ≤ 0.05, ** *P* ≤ 0.01, *** *P* ≤ 0.001, and **** *P* ≤ 0.0001.). (For interpretation of the references to colour in this figure legend, the reader is referred to the web version of this article.)

Given the observed increase in the number of blood vessels in the tissue sections with rising SC17-2 concentration, we proceeded to compare the ratio of the area stained with the vascular endothelial-specific marker CD31 to the area stained with DAPI in the same field. The view (Fig. 4A) was used to characterise the number of neovasculature. The statistical results, as shown in Fig. 4B, demonstrated that even 5 μg/mL of SC17-2 promoted blood vessel formation. Moreover, the proangiogenic effects of 10 and 20 μg/mL SC17-2 were more pronounced than that of VEGF. To further confirm the vascular promotion effect of SC17-2, a zebrafish embryonic vascular loss model was induced by 300 ng/mL VRI, and different doses of SC17-2 were added to it for comparison with an untreated zebrafish (Fig. 4D). The post-treatment of SC17-2 for 24 h resulted in a significant increase in the number of ISV in a dose-dependent manner. A 24-hour period resulted in a significant restoration of the vascular defects induced by VRI in zebrafish embryonic ISVs, with the best restoration effect observed in the 20 μg/mL SC17-2 treatment group. The number of ISVs restored reached 84.18 % of the ISVs observed in healthy zebrafish (Fig. 4C).

### SC17-2-treated HaCaT showed activation of TGF-α and TGF-β signalling pathway features

**Fig. 5:**
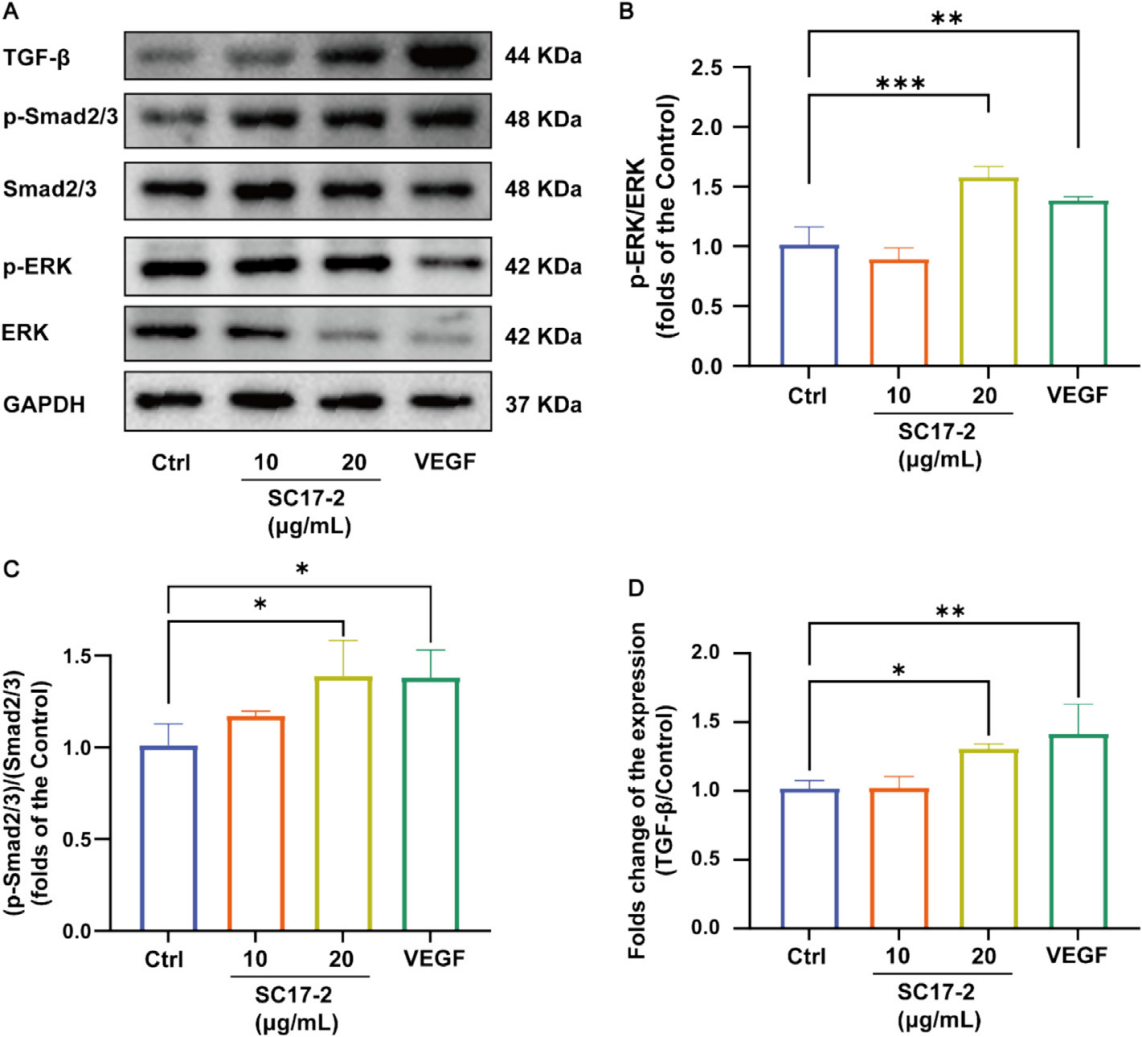
Effects of SC17-2 on p-ERK/ERK, (p-Smad2/3)/(Smad2/3), and TGF-β pathways. (A) Representative images of the five treatments on the expression levels of p-ERK/ERK, (p-Smad2/3)/(Smad2/3), and TGF-β. (B) p-ERK and ERK expression levels, 20 μg/mL of SC17-2 and VEGF treatment significantly increased ERK phosphorylation (n = 3). (C) Qualitative analysis of p-Smad2/3 and Smad2/3 expression levels, 20 μg/mL of SC17-2 and VEGF treatment significantly increased Smad2/3 phosphorylation (n = 3). (D) Quotative analysis of TGF-β expression levels, 20 μg/mL of SC17-2 and VEGF treatment was able to significantly increase the expression of TGF-β (n = 3). (Compared with the control group: * *P* ≤ 0.05, ** *P* ≤ 0.01, *** *P* ≤ 0.001).

The WB results of each pathway after treatment of HaCaT cells with 20 μg/mL of SC17-2 for 24 h are shown in Fig. 5A. The MAPK pathway plays an important role in cellular signalling, and changes in its phosphorylation level can affect a variety of cellular physiological processes, including cell proliferation, differentiation, apoptosis and stress response [42]. As shown in Fig. 5B, the increased p-ERK/ERK ratio in SC17-2-treated HaCaT cells may indicate that the MAPK pathway is activated, and the activated MAPK pathway may promote cellular activities in the wound healing process. TGF-β plays a key role in granulation tissue formation and scar formation by promoting fibroblast proliferation and collagen synthesis, thereby contributing to wound filling and scar tissue formation. Smad protein is a downstream molecule of the TGF-β signalling pathway and Fig. 5C shows that the increased phosphorylation levels of Smad protein in HaCaT cells after SC17-2 treatment and VEGF treatment may reflect increased TGF-β signalling. Increased TGF-β protein expression may be associated with the fibrotic phase of wound healing (Fig. 5D).

### SC17-2 may affect downstream signalling by affecting both conformations of EGFR

**Fig. 6:**
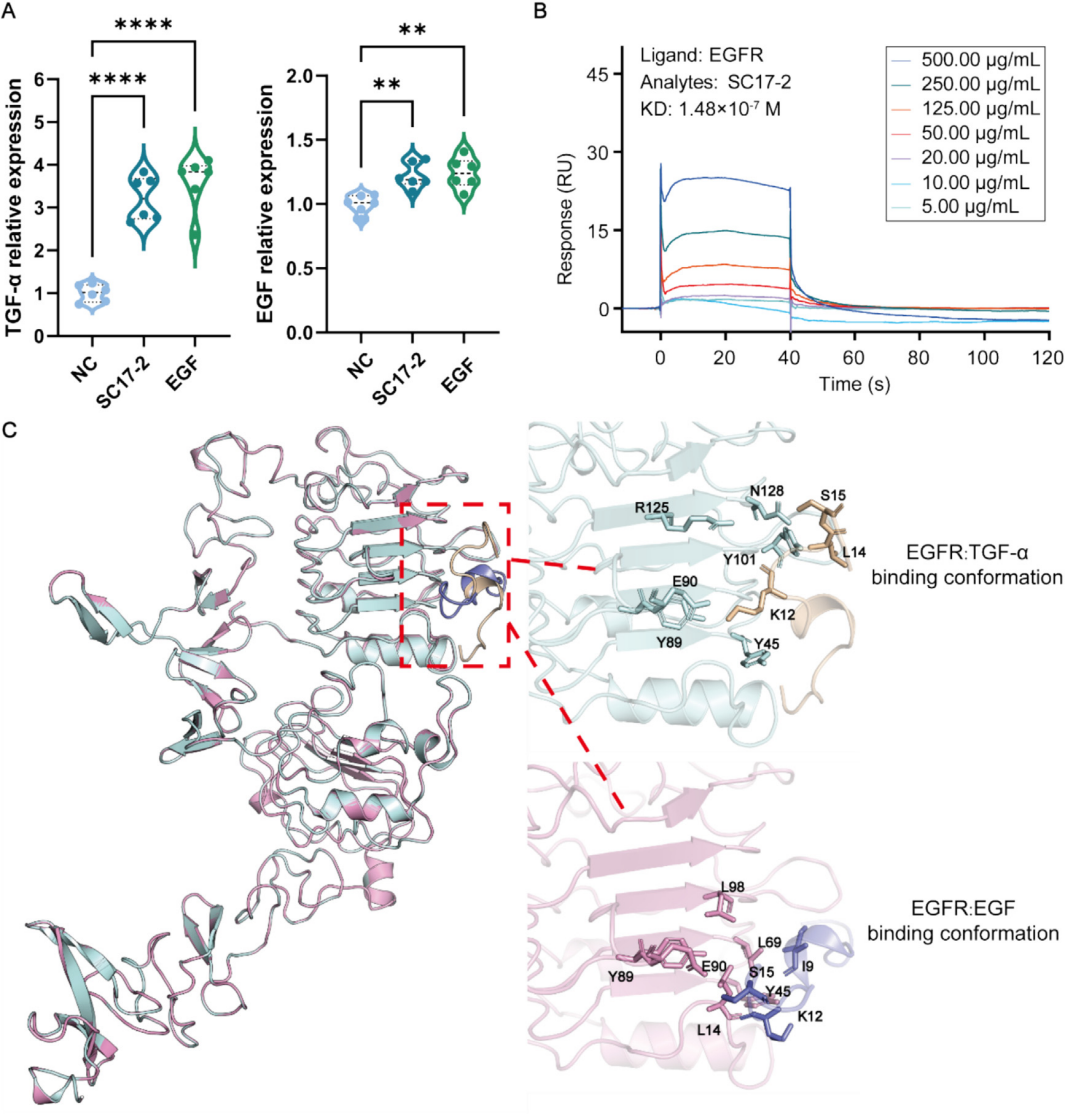
SC17-2 may bind to both conformations of EGFR, affecting the downstream expression of TGF and EGF, thereby promoting activities such as angiogenesis and cell migration. (A) mRNA expression of TGF-α and EGF was significantly elevated after treatment with 20 μg/mL of SC17-2 (n = 6). (B) SPR results showed that SC17-2 binds in a dose-dependent manner to the EGFR binding. (C) Molecular docking results showed that SC17-2 may be able to bind to both conformations of EGFR.

Due to the presence of crosstalk between the multiple signalling pathways present in the downstream of EGFR, the mRNA expression of TGF and EGF in HaCaT cells after SC17-2 treatment was also detected by q-PCR. The primer sequences employed for the amplification of TGF-α, TGF-β and EGF are delineated in Table 1. The results, as demonstrated in Fig. 6A, indicated that HaCaT cells treated with 20 μg/mL of SC17-2 exhibited a substantial augmentation in the mRNA expression of TGF and EGF in comparison to the control group, a phenomenon that was analogous to the effects observed subsequent to EGF treatment. Given the concomitant elevation of TGF and EGF expression in the aforementioned process, we sought to utilise SPR (Surface Plasmon Resonance) and BLI (Bio-Layer Interferometry) to ascertain whether SC17-2 (5–500 μg/mL) possessed the capacity to bind specifically to biotin-labelled EGFR (20 μg/mL). As illustrated in Fig. 6B, the findings demonstrated that SC17-2 exhibited a notable affinity for EGFR, with a binding constant *KD* of 1.48 × 10^−7^ M. The results of the BLI assay were consistent with those of the SPR assay, with a *KD* value of 2.29 × 10^−7^ M (as shown in Fig. S8). Subsequently, molecular docking was performed using HADDOCK, followed by visualisation using PyMOL. This demonstrated that SC17-2 was capable of binding to both the TGF-α conformation of EGFR and the EGF conformation of EGFR. This finding may provide a rationale for why several pathways mentioned in the aforementioned article were affected. In contrast, other peptides belonging to the AMP family may lack the capacity to bind to EGFR, a deficiency that can be attributed to their conformation and location (Fig. S9, Fig. S10 and Fig. S11).

**Table 1:** Oligonucleotide primers used for RT-PCR of EGF, EGFR, TGF-α.

Gene	Forward primer	Reverse primer
TGF-α	5′-CCTGCTGCCCGCCCGCCCGT-3′	5′-GCTGGCAGCCACCACGGCCA-3′
GAPDH	5′-GGCATCCTGGGCTACACTGA-3′	5′-GTGGTCGTTGAGGGCAATG-3′
EGF	5′-GCAGATGGGTCAATGCAAC-3′	5′-GGGACAGGAGCCCTTATCA-3′
TGF-β1	5′-CAATTCCTGGCGATACCTCAG-3′	5′-GCACAACTCCGGTGACATCAA-3′

## Discussion

Frogs have acquired a wide range of environmental adaptations, which can be partly attributed to the presence of AMPs, AOPs and other peptide actives in their skin [68]. Indeed, some families of AMPs may even be important for the classification and phylogenetic analyses of frogs [69]. The evolution of AMPs is continuous and dynamic [70], and as previously reported, this evolution occurs through gene duplication [71], and is subject to gene loss and pseudoprogenesis as the microbial community declines [72]. SC17-2, despite sharing a common ancestor with AMPs, lacks antimicrobial activity and has gained antioxidant, wound repair, and angiogenic activities, which represents a shift in functional focus in response to environmental changes. Our findings highlight the intricate relationship between AMPs evolution, environmental stresses, and physiological adaptations, which play important roles in clinical and other applications of AMPs.

Synergistic evolution between hosts and their associated organisms is a driving force behind the rapid and diverse evolution of molecules with relevant phenotypes and functions in organisms [73]. This phenomenon is exemplified by the evolution of immune genes related to purification selection, which are among the innate drivers [74]. The efficacy of purification selection in efficiently excluding potentially deleterious changes to amino acids in protein sequences is a key factor in this process [75]. In addition, innate immune genes must adapt to evolving microorganisms, thus maximising the diversity of related genes [76]. However, the study reported by M. A. Hanson et al. demonstrates that the evolution of innate immunity-related genes is more likely to be influenced firstly by the ecological context of the host's environment and secondly by selection pressures of specific microorganisms in the environment [72]. This explains the dynamics of AMPs and microbial communities [77], as well as why many AMPs also have additional activities and functions [33,78]. There is mounting evidence that this regulation of innate immune genes is critical for maintaining homeostasis in host-beneficial microbial communities [79,80].

The process of natural selection is a driving force behind the evolution of AMPs [71]. The interplay between different selective pressures may have led to the emergence of novel functions in AMPs [81]. The evolution of protein functional contributions is typically driven by amino acid insertions and deletions [82]. Hyphy's prediction states that purifying selection is dominant in SC17-2, where three amino acid positions (63, 66, and 73) are undergoing purifying selection. This process plays a fairly common and important role in the evolution of immune genes [76,83]. The deletion or alteration of these purifying selection sites may indicate an evolutionary trade-off or the acquisition of new functions [82,84]. For instance, the deletion of specific amino acids at positions 63 and 66 led to the elimination of the spatial site block caused by the side chain (Fig. 6C), thereby rendering SC17-2 more prone to binding to EGFR and potentially acquiring wound repair and angiogenic activities [37,85]. This deviation may be attributable to the distinctive evolutionary pressures imposed by the high-altitude environment of the Tibetan Plateau.

The process of cellular repair is facilitated by the synergistic action of multiple extracellular vesicle-associated proteins, which contribute to the immunomodulatory processes of cell proliferation, angiogenesis, and the control of the inflammatory response [86]. It is evident that EGF and TGF-α play pivotal roles in wound healing and repair processes [87]. The activation of EGFR results in an increase in TGF-α levels [88], which in turn affects downstream signalling pathways. Consequently, the produced TGF-α and EGF activate EGFR through receptor dimerization, triggering multiple cell signalling cascades [89]. The immune system, for instance, employs the evolutionarily conserved TGF-β and EGFR signalling pathways, which play pivotal roles in tissue development and wound repair [90]. TGF-β has been reported to be effective in promoting myofibroblast differentiation and restoring the vascular barrier during wound repair [91]. This is primarily due to the elevated ratio of phosphorylated Smad 2/3 in the TGF-β/Smad pathway and the production of collagen [92], accelerating wound repair, which is consistent with the trend of our western blot experimental results (Fig. 5).In addition, we simulated the regeneration of vascular destruction under pathological conditions by VRI-induced vascular injury model in zebrafish likewise demonstrated the vascular repair activity of SC17-2 [93]. EGFR activation by different ligands leads to varying cellular responses [94], including enhanced cell migration [95] and angiogenesis [96] he reduced microbiome diversity (Fig. 6A). qPCR results showed a simultaneous increase in TGF-α, EGF and TGF-β1 (Fig. S6A and Supplementary Material Fig. S7) expression due to a complex crosstalk between EGFR, TGF-β, and VEGF, as evidenced by an increase in VEGF with the up-regulation of EGFR signalling [97], which resulted in the activation of downstream ERK in response to cell proliferation and migration(Fig. 5B) [98].

EGFR is capable of being activated by seven different growth factors and is therefore involved in the regulation of many key cellular programmes [99]. Interestingly, this regulation through EGFR shows diverse and differential downstream signalling regulation with different ligands [94]. The increase in EGF and TGF-β after SC17-2 treatment observed in this study appears to substantiate the existence of these multiple regulatory mechanisms. In addition, EGFR *in vivo* has been shown to adopt both oligomeric [100] and dimeric [101] structures, with both forms being active in transforming small directional changes in the transmembrane region into different active forms [37,102].

In addition to this, conformational differences in the binding of different ligands to the same structure of EGFR appear to explain the downstream signalling differences [37], and Mozumdar D et al. demonstrated the tuning of the differential EGFR signalling output by mutationally switching the conformation of near-membrane fragments [103]. As demonstrated in Fig. 6C, this differential difference was clearly evident in the Docking results. SC17-2 has been demonstrated to bind to Y101, N128, N128 and R125 on EGFR, thereby functioning as a TGF-α through signalling. Furthermore, EGF-specific binding sites L98, L69 and I9 can also be bound by SC17-2. This may provide a rationale for its function in cell migration, angiogenesis, and the activation of downstream pathways, including ERK and Smad2/3 phosphorylation. The pleskein family of AMPs have been reported to have tissue repair activity [13], and, as can be seen in Fig. 1A, the pleskein family all have some missing amino acid sites. The spatial location of other AMPs means they do not match well with the 'C-helix' region of EGFR (Supplementary Material Fig. S9, Fig. S10 and Fig. S11)[104]. The majority of these missing amino acid sites have been shown to be Purifying selection, and thus the elimination of these purification selection sites may be an adaptation to the stress selection present at high altitudes in the Tibetan Plateau [105].

The Tibetan Plateau, with an average altitude of 4000–5000 m [106], is characterised by extreme and harsh environmental conditions, including low temperatures and intense ultraviolet radiation [107]. These conditions have been shown to exert significant ecological stresses on local microbial communities, leading to a reduction in microbial diversity and abundance [108]. The reduced microbiome diversity in this environment has significantly influenced the composition and function of skin-active peptides in *N. parkeri*. In such an environment, peptide SC17-2 may not need to rely excessively on the traditional functions of AMPs, but rather respond to intense UVB stress by, for example, eliminating free radicals. In addition, its potential effects on the vascular system may give it a survival advantage in cold environments.

The results of the positive selection analyses indicated that strong positive selection occurred primarily in the mature peptide portion of the AMPs (Fig. 1A). Furthermore, this strong positive selection within the mature peptide in the AMPs at high altitude was also observed in other species of frogs (*Rana temporaria* and *Rana arvalis*) [23]. Finally, it was determined that highly variable mature peptide structural domains were the result of adaptation to the challenge of pathogens, leading to an increase in the genetic diversity of the AMPs [109]. This study hypothesises that the genetic diversity of AMPs may be attributable to the unique ecological niche of low-level environmental microorganisms within the plateau environment. A parallel can be drawn with AMPs isolated from other plateau frogs, such as *Odorrana tiannanensis* [110], which also exhibited reduced antimicrobial activity. The observation of positive selection and diminished antimicrobial activity (or even the absence of such activity [13]) in response to the plateau environment suggests a potential functional transformation of AMPs.

*N. parkeri*, an endemic frog species, has adapted to the extreme environment of the Tibetan Plateau through unique modifications in its skin-active peptides [3]. This study aims to elucidate the mechanisms by which *N. parkeri* diversifies through the frog-specific AMPs family to enhance environmental adaptation. However, this study is not without its limitations. Firstly, the activity of only one AMPs, SC17-2, was verified, and the wound repair and antioxidant activities of other AMPs families in *N. parkeri* were reported in other articles. Secondly, due to limitations in research time and other factors, the reason for the differences in signal transduction came from indirect evidence such as western blot experiments and molecular docking. ToxinPred indicated that SC17-2 was non-toxic and HemoPI2.0 predicted an HC_50_ of 77.22 μg/mL, which far exceeded the concentration used in the test. It is also worth noting that AllerTOP indicated that SC17-2 may cause an allergic reaction. Further experiments are required to confirm the detailed research results.

## Conclusion

The present study elucidates the unique evolutionary trajectory and functional divergence of SC17-2, a skin peptide from the high-altitude frog *N. parkeri*. Contrary to traditional AMPs, SC17-2 exhibits redundant functions, re-emphasising the pivotal role of ecological background over specific microbial selection pressures in shaping its evolutionary adaptation. In the harsh environment of the Tibetan Plateau, SC17-2 has evolved distinct angiogenic and cell migration-promoting activities, which are distinct from those of conventional AMPs. This functional divergence underscores the adaptability of peptides in response to environmental challenges and highlights their potential in regenerative medicine. The study's findings challenge the conventional paradigm of AMPs being primarily antimicrobial, advocating for a broader appreciation of their roles in ecological contexts. The substantial promotion of wound healing and angiogenesis exhibited in both mouse and zebrafish models indicates the potential for SC17-2 to have therapeutic applications. Such investigations could lead to the development of novel therapeutic strategies and enhance our understanding of peptide evolution in extreme environments.

## Compliance with Ethics Requirements

All Institutional and National Guidelines for the care and use of animals (fisheries) were followed.

## CRediT authorship contribution statement

**Kaixun Cao:** Conceptualization, Methodology, Formal analysis, Writing – original draft. **Liting Zhang:** Conceptualization, Methodology, Investigation, Formal analysis, Writing – original draft. **Min Yang:** Methodology, Validation, Investigation. **Jinai Gao:** Visualization, Methodology. **Congshuang Deng:** Visualization. **Xiaoshan Huang:** Data curation. **Qian Chen:** Methodology. **Qiumin Lu:** Methodology. **Yiji Cheng:** Methodology. **Shaoyang Gao:** Methodology. **Hui Cao:** Conceptualization, Supervision, Writing – review & editing. **Ren Lai:** Conceptualization, Supervision, Writing – review & editing, Funding acquisition.

## Declaration of competing interest

The authors declare that they have no known competing financial interests or personal relationships that could have appeared to influence the work reported in this paper.
