# Supplementary material for: Adaptation of plateau frog peptide: From antimicrobial to angiogenic and proliferative functions
Source: J Adv Res. 2025 Jun 7;81:287–300. doi: 10.1016/j.jare.2025.06.013 (PMC12957798; doi:10.1016/j.jare.2025.06.013)
Supplement: Supplementary Data 1 [file mmc1.docx]

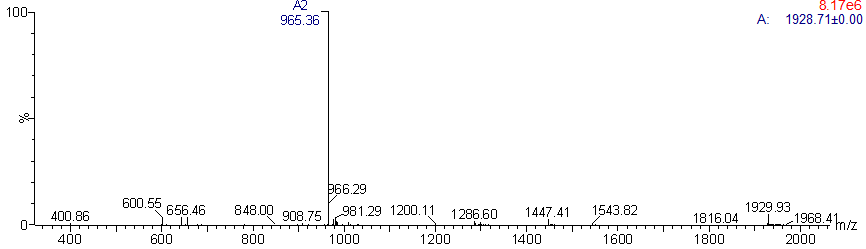


Figure S1. HPLC infographic of SC17-2 synthase.


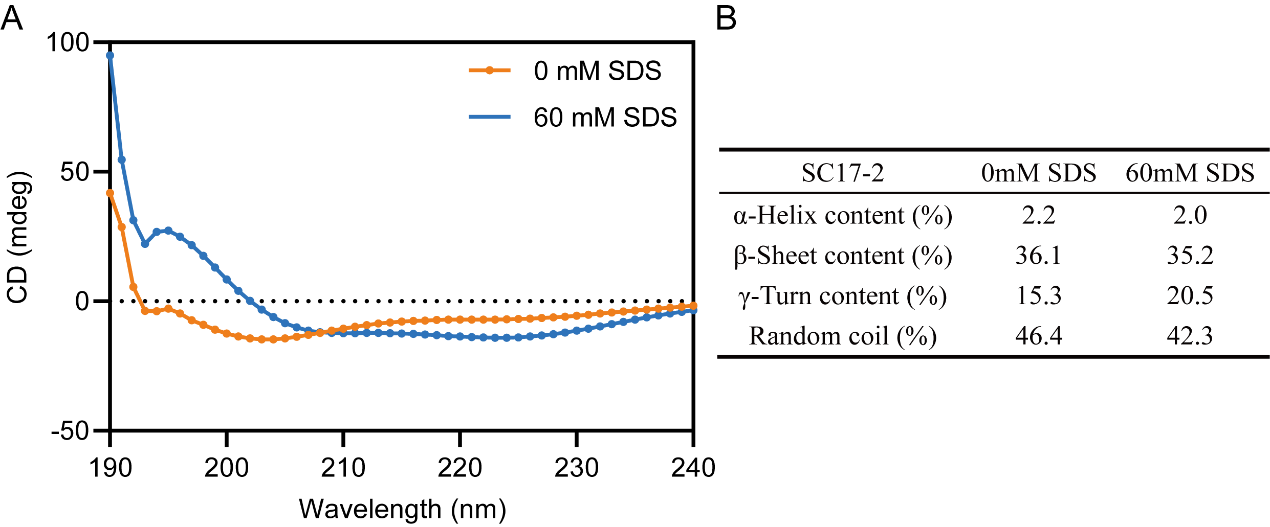


Figure S2. Circular dichroism spectral results of SC17-2 in 0 mM (H2O) and 60 mM SDS.


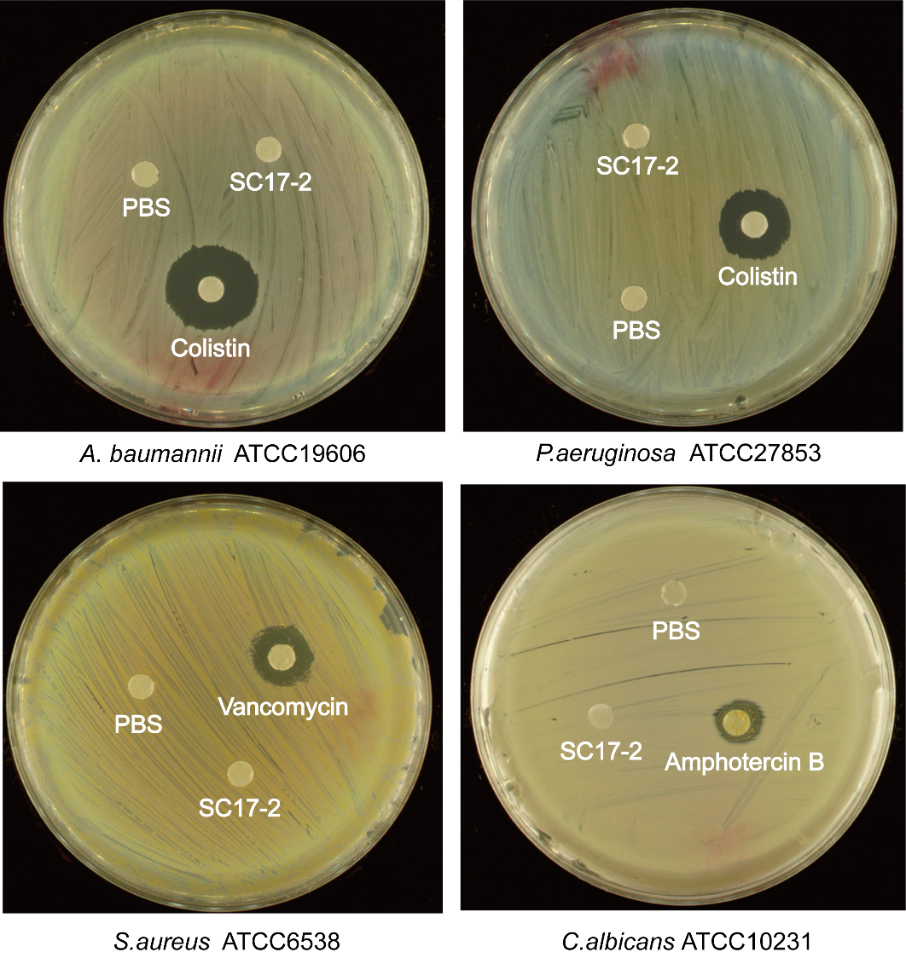


Figure S3. The results of paper diffusion assay showed that SC17-2 (10 mg/mL) had no bacteriostatic activity.

Table S1. The MIC of SC17-2 was determined through experimentation with three type strains of bacteria.

| Microbiological name | SC17-2 (μg/mL) | | | | | | | Vancomycin | Colistin |
| --- | --- | --- | --- | --- | --- | --- | --- | --- | --- |
|  | 0 | 3.125 | 6.25 | 12.5 | 25 | 50 | 100 |  |  |
| *Escherichia coli*  ATCC2771 | NS | NS | NS | NS | NS | NS | NS | - | 0.78 |
| *A. baumannil* ATCC1968 | NS | NS | NS | NS | NS | NS | NS | 0.78 | - |
| *S. aureus* ATCC6538 | NS | NS | NS | NS | NS | NS | NS | - | 0.78 |


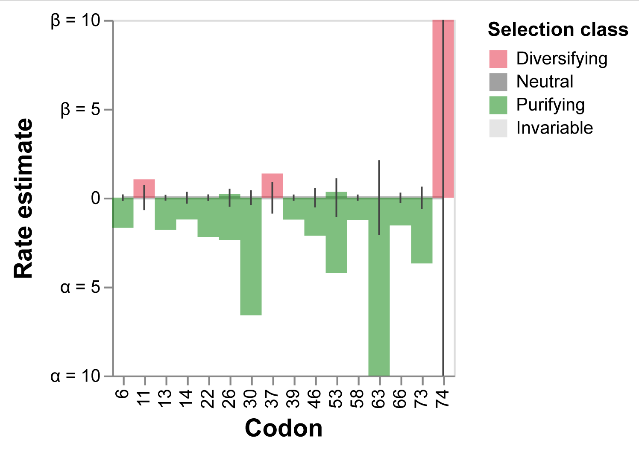


Figure S4. Maximum likelihood estimates of synonymous (α) and non-synonymous rates (β) at each site shown as bars. The line shows the estimates under the null model (α=β). Estimates above 10 are censored at this value.


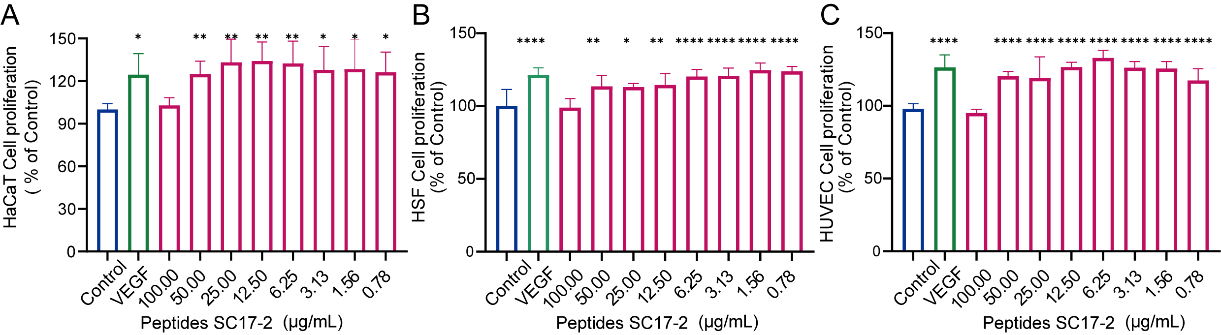


Figure S5. Results of a gradient concentration of SC17-2 based on the CCK-8 method 16 h after the action of HaCaT (Human Keratinocytes Cells), HSF (Human Skin Fibroblast) and HUVEC (Human Umbilical Vein Endothelial Cells).


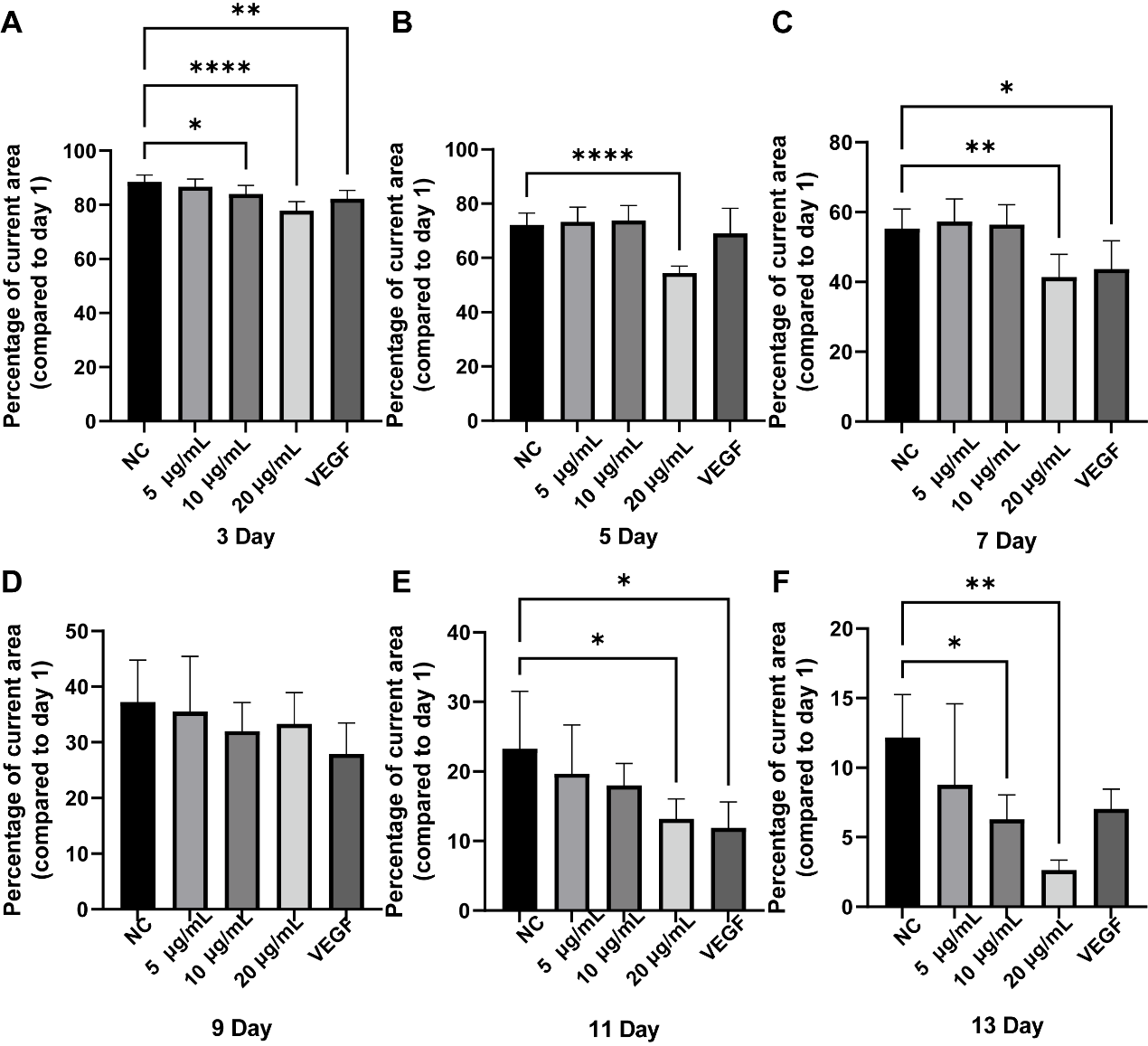


Figure S6. Wound area between treatments between days 3 and 13 compared to day 1 (%).


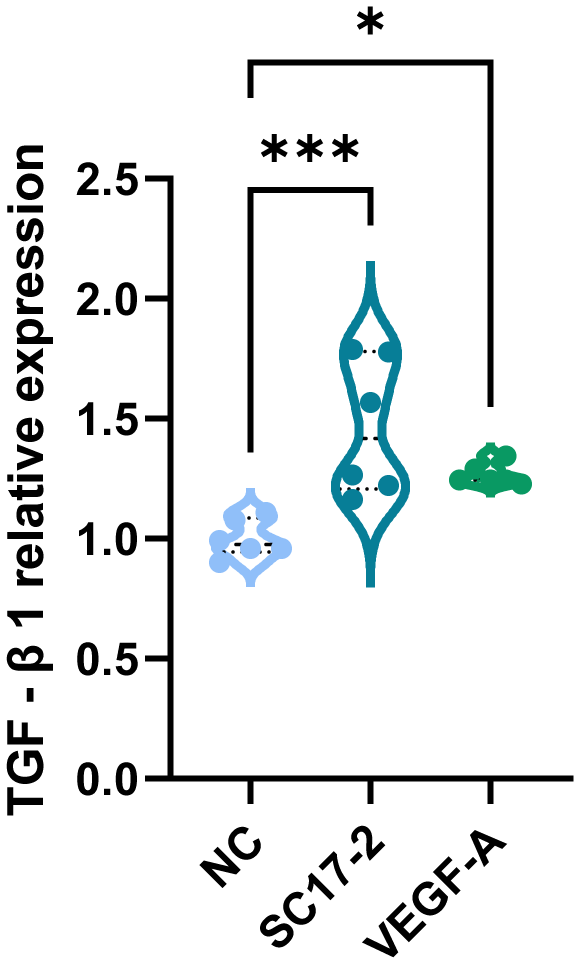


Figure S7. qPCR quantification of TGF-β1.


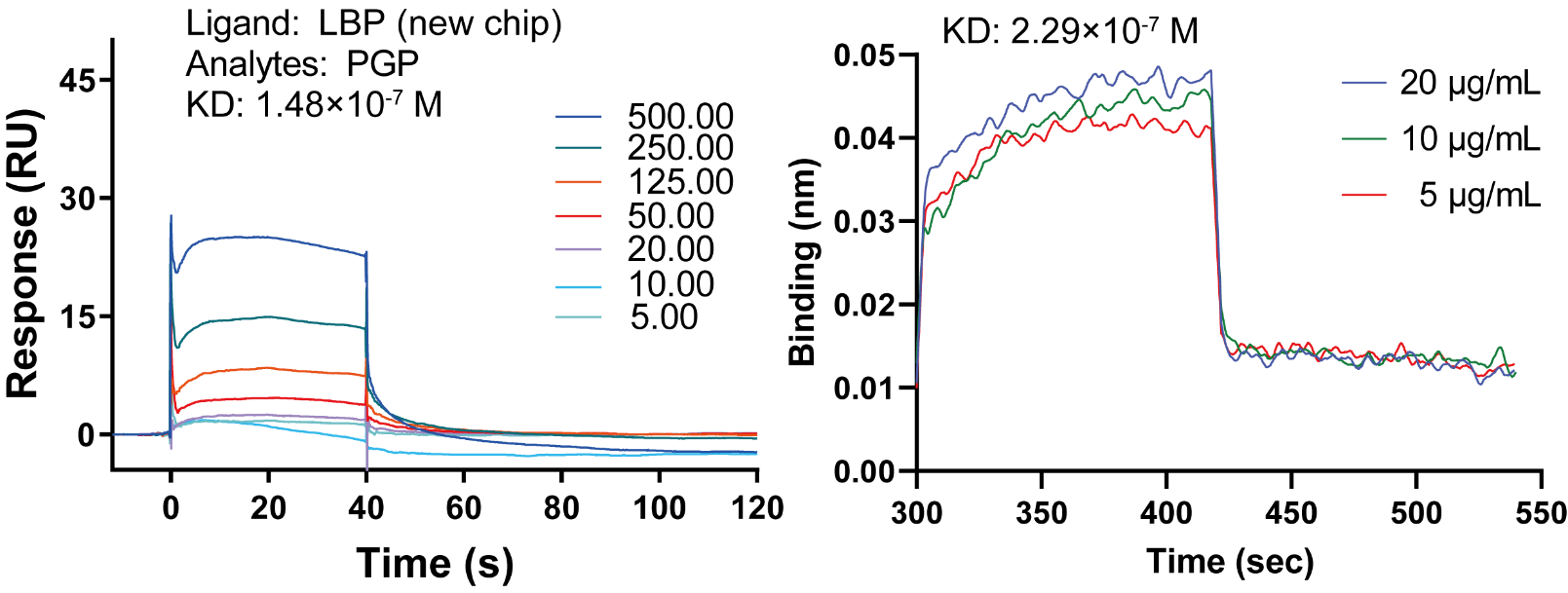


Figure S8. BLI results showed that SC17-2 binds in a dose-dependent manner to the EGFR binding.


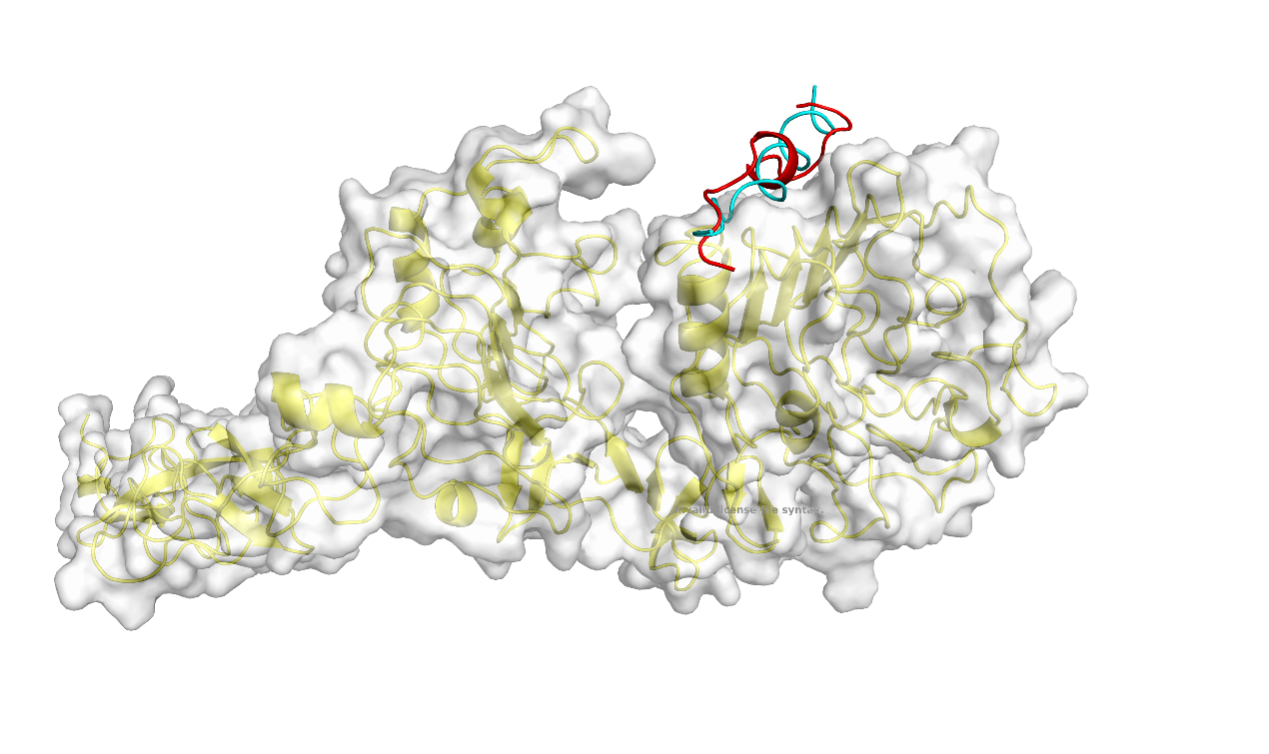


Figure S9. Spatial location of AIU999321 in relation to EGFR.


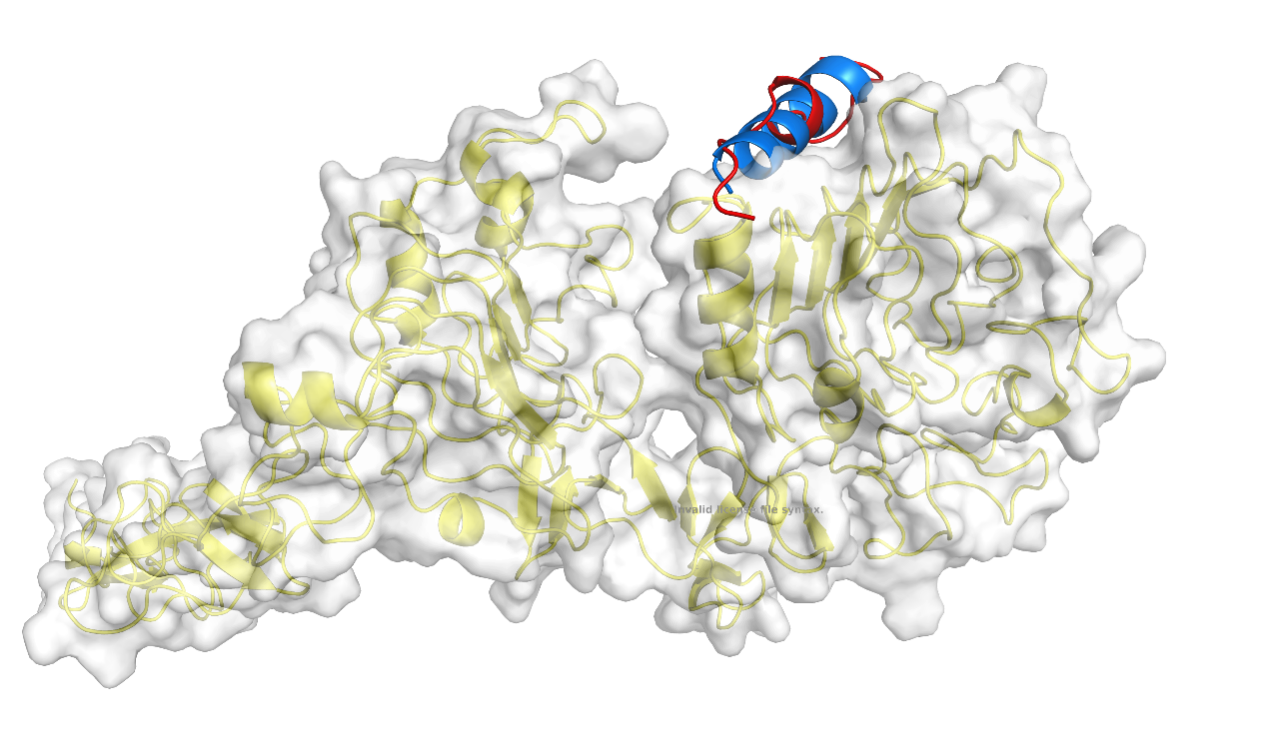


Figure S10. Spatial location of AIU999051 in relation to EGFR.


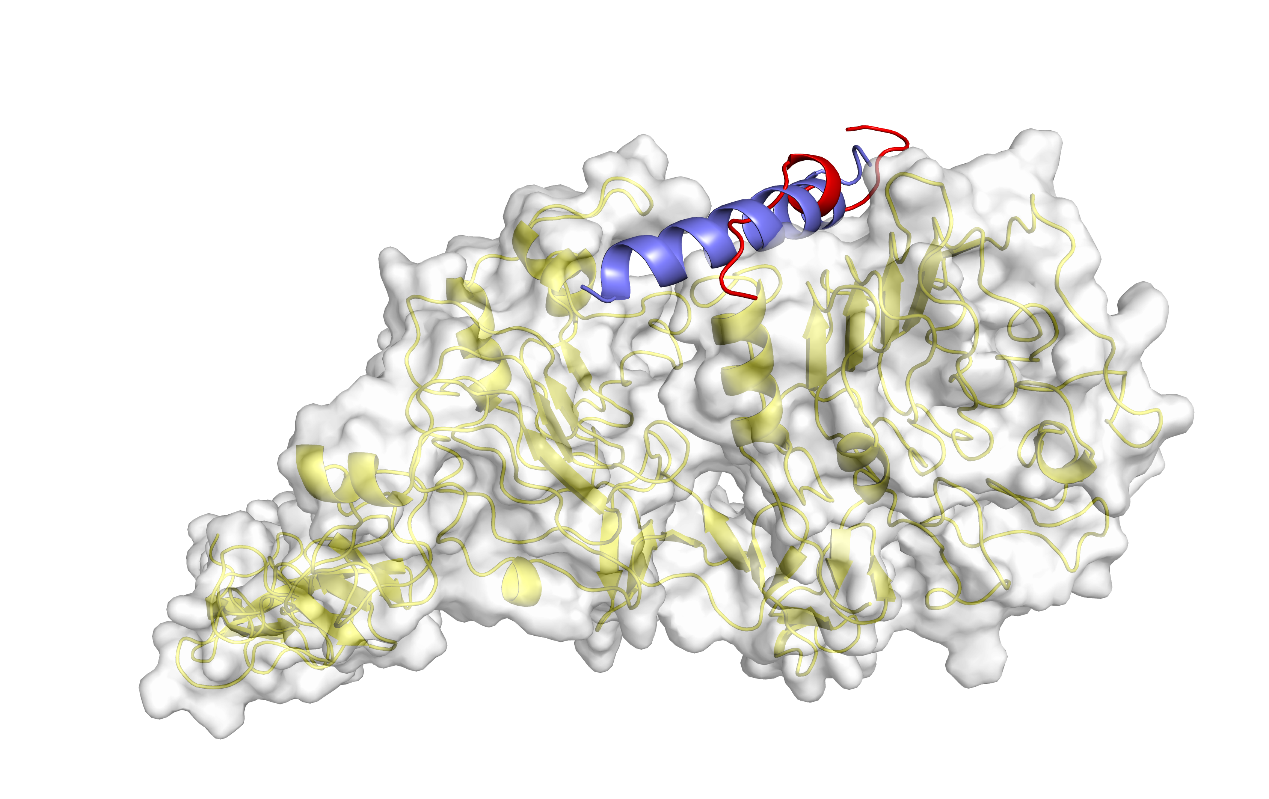


Figure S11. Spatial location of AIU998731 in relation to EGFR.
